# Historical Surgical Treatments in Polish Veterinary Medicine

**DOI:** 10.3390/ani10091487

**Published:** 2020-08-24

**Authors:** Slawomir Gonkowski, Liliana Rytel, Krystyna Makowska, Jaroslaw Calka

**Affiliations:** 1 Department of Clinical Physiology, Faculty of Veterinary Medicine, University of Warmia and Mazury, Oczapowskiego Str. 13, 10-718 Olsztyn, Poland; jaroslaw.calka@uwm.edu.pl; 2 Department of Internal Medicine with Clinics, Faculty of Veterinary Medicine, University of Warmia and Mazury, Oczapowskiego Str. 13, 10-718 Olsztyn, Poland; liliana.rytel@uwm.edu.pl; 3 Department of Clinical Diagnostics, Faculty of Veterinary Medicine, University of Warmia and Mazury, Oczapowskiego Str. 13, 10-718 Olsztyn, Poland; krystyna.makowska@uwm.edu.pl

**Keywords:** veterinary history, Poland, surgical operations, bloodletting, setons

## Abstract

**Simple Summary:**

This review describes the most popular methods used for treating animals in Poland from the 16th to the 20th century described in Polish veterinary literature. Bloodletting, setons, ‘fonticulus’, cauterizations, injections and other former veterinary methods are described. This review presents the development of veterinary medicine from ancient cures to a modern branch of science.

**Abstract:**

Several methods of surgical treatments have been used in the history of Polish veterinary medicine, many of which have now been forgotten. In the present study, a review was conducted of Polish-language veterinary medicine books published from the 16th century (when the first books in Polish were printed) to the 20th century. The article contains a description of the most popular surgical methods used in animal treatment in Poland over the centuries including, among others, bloodletting, setons, fonticulus and cauterization. This article reviews historical veterinary methods and traces the development of Polish veterinary medicine from ancient cures often based on humoral theory to a modern branch of biologic science.

## 1. Introduction

The first written source concerning animal treatment in Slavic lands is from the fourth century A.D. At that time, Apsyrtus—a veterinary surgeon in the Byzantine army during a military campaign came into contact with Slavic warriors and noted that they treated horses suffering from urinary retention by smudging the animal with the smoke from a secretion from beaver glands [1,2].

Further development of Polish veterinary medicine continued in the Middle Ages. It is known that in the 13th century there were falconers and dog handlers at the Polish royal court, and they were probably also responsible for treating the animals under their care [3]. Nevertheless, for many ages, the main animal species of interest to veterinary care was the horse, and the treatment of this species was often left to blacksmiths. The first person known by name who dealt with the treatment of animals was the blacksmith Jakusz [1]. He lived at the turn of the fourteenth and fifteenth centuries and treated the horses of King Władysław II Jagiełło. Over time, blacksmiths who treated horses came to be called “konował” or a “person fluent in the art of animal treatment, who dealt not only with shoeing the horses, but also with the treatment of animals”. Over time the term “konował” was identified with “veterinarian” and it was used until the beginning of the nineteenth century [1]. Interestingly, in modern Polish the word “konował” means “a bad doctor” or “quack”.

**Figure 1:**
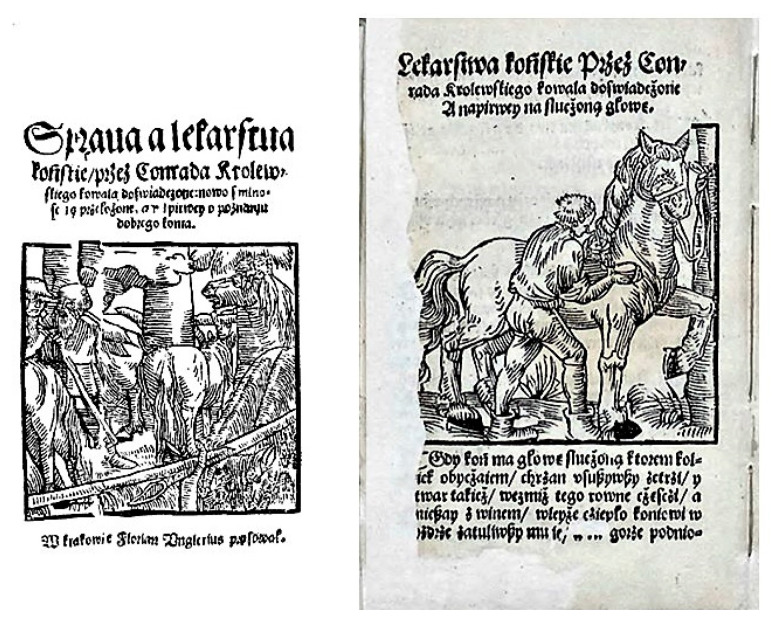
(**left**) Title page of the first Polish-language veterinary book published in 1532 and (**right**) an illustration from this book showing ointment administration.

The first books on issues related to animal treatment printed in Polish were published in the 16th century [1]. The first of them was a small, anonymous work entitled, “The matter of horse medicines…” (title in Polish: “*Sprawa a lekarstwa końskie…*”) published in 1532 and containing a description of symptoms and treatments of several of the most commonly found horse diseases (Figure 1). From that time onward, veterinary knowledge became available to people who did not understand Latin.

The present work describes the most popular surgical methods used in animal treatment in veterinary books printed in Polish from the 16th century to the 20th century.

## 2. Bloodletting

Bloodletting is probably the most popular surgery in the history of medicine and veterinary medicine. This method of treatment was known in the ancient Egyptian, Greek and Roman medicine and was used in therapies of humans and animals. According to legend, the inventors of this method of treatment were Egyptian sacred hippos, who did bloodletting with harp papyrus reed when they became too fat from too much food [4]. It should be pointed out that bloodletting was commonly used in medicine and veterinary medicine for many years, from ancient times up to the 20th century.

The oldest historical evidence that bloodletting was applied in the territory of Poland dates back to the late 11th century. It involves the discovery of a bloodletting knife which was found on the grounds of an early medieval Slavic hill fort located in Bródno (at present, it is the part of Warsaw—the capital of Poland) [4]. It is not known whether this device was used by a medical doctor or a person treating animals, but it is proof that bloodletting was known in Poland in the early middle ages.

Bloodletting was described in almost all Polish veterinary medicine books, which were published from the 16th century to the first half of the 20th century. In the above-mentioned first Polish veterinary book printed in 1532, the anonymous writer recommended bloodletting during eye diseases with the words “*cut the veins above both eyes. Let the blood flow out*” and after head contusion by the phrase: “*to open the vein on the neck*” [5]. Bloodletting was also used during laminitis (not only on the veins in the legs, but also on the neck), edema, cough and various types of inflammatory processes [5].

The next book printed in Polish language in the 16th century, in which aspects connected with veterinary medicine were included was “Books about holding” (title in Polish: “*Księgi o gospodarstwie…*”) written by Pietro de’ Crescenzi (a medieval Italian surgeon and naturalist), translated to Polish by the royal secretary Jędrzej Trzecieski and published in 1542 (the second edition in 1571) [1]. In this book, (which was relatively rational and free from superstition, compared to other veterinary publications in this period), bloodletting was recommended in horses during asthma, urinary retention, “*impotence in the chest*” (probably pneumonia and bronchitis), “*cold of the head*” (probably connected with inflammatory processes within the tissues at the head) and strangles [6]. Moreover, bloodletting was prescribed in cows and sheep during distension and fever of the head. In the latter case, the veins under the tongue were pierced using the sharp spikes [6]. In turn, the book “An experienced medicine” (title in Polish: “*Lekarstwa doświadczone…*”), written by Marcin Siennik and published in 1564, recommended bloodletting in horses during lameness [7].

Bloodletting was also very popular in Polish veterinary medicine of the 17th and 18th century. Jan Ostroróg in “The hunting with hounds” (title in Polish: “*Myślistwo z ogary*”)—the first Polish book entirely devoted to breeding and treatment of dogs, which was published for the first time in 1608 [1,3], recommended bloodletting during rabies (in words: “*bloodletting in rabies is the base of the treatment*”), as well as in the cases of leg swelling [8]. The using of bloodletting was also recommended by Krzysztof Dorohostayski in his book “Hippica. Books about horses” (title in Polish: “*Hippika to jest o koniach księgi*”, first published in 1603), which is a comprehensive description of the breeding, treatment and use of horses [1]. Dorohostayski advised bloodletting for a wide range of horse diseases including, among others, blindness, hypertrophy of the third eyelid, laminitis, internal inflammation and “*tremor of the heart*” (unspecified diseases with symptoms from the cardiovascular system) [9]. Moreover, the author described the close relationships between bloodletting, astrology and superstition. He wrote that the success of the treatment depends on the moon phase, as well as the constellations of the planets and stars. For example, Dorohostayski claimed that bloodletting cannot be performed on specific days, such as on January 3, 4, 5, 9, 13, February 13, 17, 19, March 13, 15, 16, etc. He also mentioned “*Egyptian days*”: “*anniversary of days, when the God visited the plagues on the Egyptians*” and any treatment started on those days would fail [9]. Dorohostayski categorically prohibited bloodletting on a new or full moon because this operation at that time could result in the death of animals. “Hippica…” mentioned more than 30 various veins, in which bloodletting may be done and the choice of the vessel depended on the disease and the time of the year. For example, according Dorohostayski, in the spring or autumn, bloodletting should be done on the cervical veins, in the summer—on the veins of legs or chest and in the winter on vessels on the sides or abdomen of animal [9]. Bloodletting was specifically recommended by Dorohostayski during “*windy laminitis*” by “… *taking thick sticks, putting them into the horse nostrils and spinning them as long as they bleed*” [9].

Bloodletting was also popular in Polish veterinary medicine throughout the 18th century. All books concerning the animal treatment and farming printed at that time recommended this operation both as therapy during various diseases as well as a method of immunization of healthy domestic animals of all species. For example, the book “Horse pharmacy” (title in Polish: “*Apteczka końska*”) printed in 1794 described bloodletting to cure headache in horses with the words: “*The first and most effective treatment against this disease is to perform bloodletting as soon as possible to free the vessels from unnecessary clogging*” [10]. The book “The work of house-keepers and peasants” (title in Polish: “*Dzieło doręczne dla ekonomów i wieśniaków”)* printed in 1800 states that “*In May, bloodletting from the vein under the tongue should be done in all cows, because it is great help against the plague*” [11]. In the 18th century and the first years of the 19th century, bloodletting was also used in diagnosis. For example, if the blood was “*black and dry*” it may have indicated the inflammatory processes, and if the blood was watery, it indicated “*rot within internal organs*” [10].

**Figure 2:**
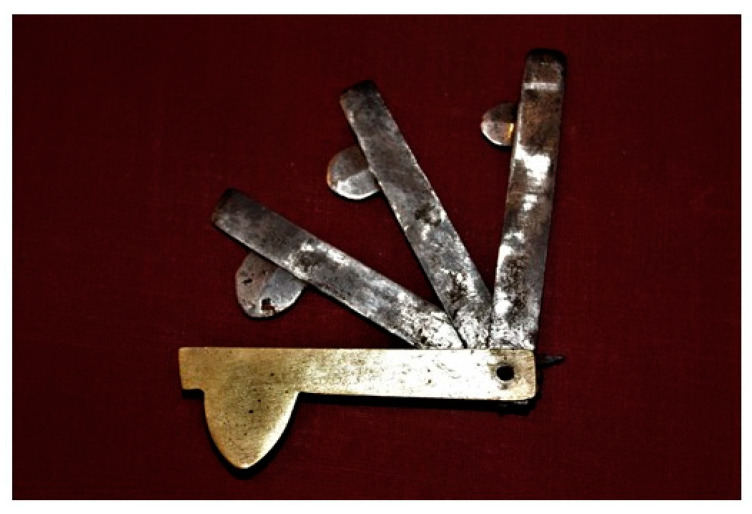
Bloodletting fleam from the 19th century from the private collection of S. Gonkowski (photo. L. Rytel).

In the 19th century, there was significant progress in biologic and medical sciences. However, this development did not cause a decrease in the popularity of bloodletting. The precise description of this method can be found in “Practical veterinary surgery” (title in Polish: “*Chirurgia weterynaryjna praktyczna*”) written by Edward Ostrowski and published in 1845, which is a true treasury of knowledge concerning 19th-century veterinary practices. Ostrowski mentioned bloodletting as a simple surgical operation and recommended it for “*all acute fevers, inflammatory diseases, external inflammatory, multiplicity of blood into noble internal organs, including heart and brain, weakened activity of the brain and nerves*” [12]. He also recommended bloodletting to fatten animals, as well as to increase the likelihood of insemination in mares. Ostrowski described the exact method of bloodletting. To conduct bloodletting, the veterinarian should “*track down the vein*”, place a bloodletting fleam (Figure 2) on the vein and hit the blade with the wooden baton (a small hammer). After the hit and opening the vein, the bloodletting blade should be immediately withdrawn. To increase the blood flow, the vein should be pressed with the fingers. For bloodletting from vessels located on the neck, the blood flow was increased by a cord loop tightened around the neck above the place of bloodletting. During bloodletting, the blood was collected in the bowl with a scale to determine the amount of blood flowing out from the vein. Ostrowski wrote that the amount of blood released during one bloodletting should not exceed 6–12 pounds in cattle, 5–10 pounds in horses, 1 pound in big pigs, 1/4 to 1/2 pound in sheep and goats and 1/6–1/2 pound in dogs [12]. At the end of bloodletting, the incision of the vein was secured with thread or horsehair.

Apart from the typical bloodletting curried out with bloodletting blades, Ostrowski also described two kinds of “*local bloodletting*” [12]. One of them was an incision also called “*scarification*”, which consisted of cutting small vesicles under the skin using an apparatus known as a scarificator. Such treatment was used during some skin lesions and diseases with edema. The second type of “*local bloodletting*” included leeches and cupping therapy. According to Ostrowski, these methods were too expensive in the treatment of large animals. Therefore, he recommended them only in small animals (dogs and cats), but, in his opinion, leaches and cupping therapies could also be replaced by “typical” bloodletting [12].

**Figure 3:**
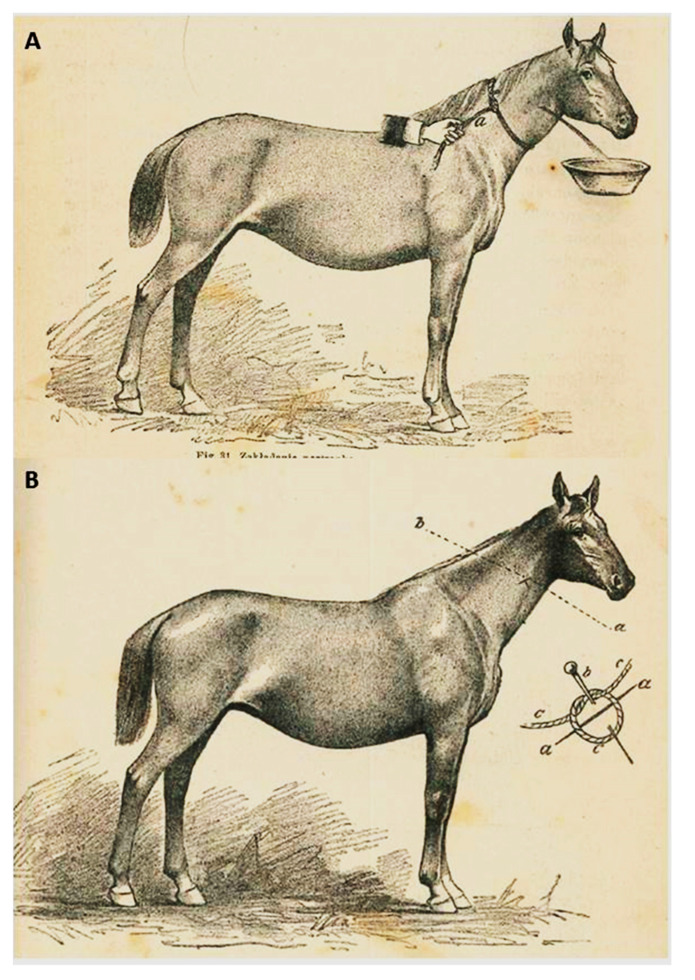
(**A**) Bloodletting and (**B**) the manner of vein securing after bloodletting. Illustrations from the book, “Domestic animals in health and disease” published in 1912.

The popularity of bloodletting in the 19th century can be demonstrated by the fact that the book “Manual of household veterinary” (title in Polish: *Poradnik weterynaryi gospodarczej*”)—written by Jakub Henryk Lewandowski and published in 1858—was one of the most popular veterinary Polish books at that time and it recommended bloodletting for seventeen out of forty described horse diseases [13]. At the turn of the 19th and 20th centuries, bloodletting in Polish veterinary medicine became less popular, but it was still done (Figure 3).

Veterinary books from that period recommended bloodletting during laminitis, meningitis and “*lung congestion*” (blood influx into the lungs), as well as scarification during diseases with edema [14,15,16]. However, books published at the turn of the 19th and 20th centuries (contrary to earlier times) did not recommend this operation as a method of immunization in healthy animals. The book “Popular veterinary for rural householders” (title in Polish: “*Weterynaria popularna dla gospodarzy wiejskich*”) published in 1893, states that “*bloodletting as a precautionary measure is of no value, and the use of bloodletting for this purpose is a superstition*” [14].

The last Polish veterinary medicine books describing bloodletting as a method of treatment (for laminitis) were published just after World War II [17]. Nevertheless, it cannot be excluded that bloodletting continued to be performed over the next years in the Polish countryside, not by educated veterinary surgeons, but by quacks and owners of animals. Interestingly, at the present, bloodletting is still carried out in human medicine, but the use of this method is limited to specific diseases, including hemochromatosis, polycythemia vera and porphyria cutanea tarda, in which a reduction in red blood cells is desirable [18,19].

The widespread use of bloodletting over the centuries is probably the result of the theory of humoral medicine, according to which health depends on the balance between four organic humors (temperaments): blood, yellow bile, black bile and phlegm and it was believed that bloodletting restored this balance. In most cases, bloodletting was harmful to the patient and resulted in body weakness, but during some diseases connected with increased blood pressure, as well as in sunstroke or acute inflammation (for example in laminitis), such treatment often relieved sick animals.

## 3. Setons

**Figure 4:**
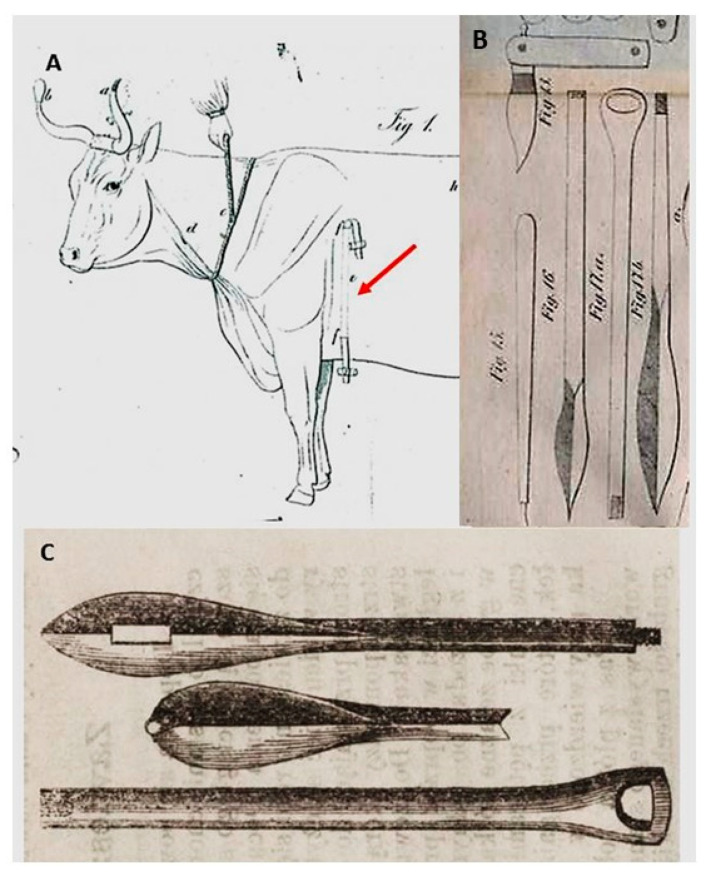
(**A**) Seton (indicated with an arrow). Illustration from “Practical veterinary surgery” (1845) written by Edward Ostrowski; (**B**) seton needles and bistoury. Illustration from “Manual of household veterinary” (1858) written by Jakub Henryk Lewandowski; (**C**) seton needles: (top and bottom) two-part French needle with two eyes and (center) English needle with a button. Illustration from the journal “Biblioteka Rolnicza” (1875).

Setons is a surgical treatment consisting of putting a cord, horsehair or linen through the fistula between the skin and muscles to relieve chronic diseases and/or abscessation (Figure 4A).

In the history of Polish veterinary medicine, setons were equally popular as bloodletting. They were mentioned by all Polish-language veterinary books published from the 16th century to the first half of the 19th century. For example, the book “The matter of horse medicines…” in 1532 recommended setons during the treatment of horse glanders [5]. The operation was conducted on the base of the tail and consisted of the separation of the skin from the muscles by a red-hot brazen rod and putting a piece of goat tallow-soaked string in the wound. The string had to be changed daily and treatment was repeated until the condition of the animal improved. Another 16th century book, “Books about holding” described setons on the chest or under the throat done with horsehair or tow for treating strangles [6].

Over the centuries, the techniques of making setons, the tools needed, and the treated diseases have changed. In the 16th, 17th and the first half of the 18th only sharp knives, bistours, sticks and metal rods (often heated) were used to separate the skin from the muscles [5,8,9]. In the 19th century, special seton needles were constructed (Figure 4B,C). In Polish veterinary medicine books and journals published in the 19th century, the following types of seton needles were mentioned [12,20]: German needles—dull with one eye, often-folding, French needles—sharp with two eyes on two opposite ends and English needles—with a button and crescent-shaped with sharp end needles—only for setons on a hoof frog.

The most accurate description of setons can be found in the book “Popular veterinary surgery” printed in 1845 [12]. According to this book, setons were employed as follows: (1) the veterinarian grabbed the skin, formed a fold and cut the skin with the bistoury; (2) he then put the seton needle with a strap into the incision and separated the skin from the muscles over a distance of 10–12 inches, (3) the second fold of the skin was formed (4) the second incision of the skin (on the second fold) was made and the needle was removed through the second hole, (5) a strap was tied to avoid moving it under the skin. The above-mentioned technique of setons was typical for large animals (horses and cows). In small animals (dogs) only one fold of the skin was formed, and it was pierced with the sharp needle. The strap was replaced by a short thin (most often woolen) thread.

Very often, irritant substances were used to strengthen the setons [13,21]. The skin and the place of incision were rubbed (for example, with powdered brick) “*to make it easier to separate the skin from the muscles*” [22] and the strap was soaked with Spanish fly (*Lytta vesicatoria*), turpentine or the root of Veratrum nigrum in vinegar [12]. The strap was left under the skin for two or three weeks [12,20,21]. When it was removed, the wounds after incisions were left to heal.

In the history of Polish veterinary medicine, setons were primarily used during inflammatory processes, infectious diseases, paresis and diseases with high fever [12,21], but also as a vaccination method for healthy animals during plagues. Such use of setons during a “*cattle plague*” is described in the book “The work of house-keepers and peasants” published in 1800 [11]. Interestingly, this book recommended that setons should be done in the parts of the body of healthy animals which were pathologically changed in sick cows. The exact descriptions of the purposes for which setons were made can be found in the above-mentioned “Popular veterinary surgery” [12]. The author of this book (Ostrowski) mentioned four aims of setons in animal treatment, namely: (1) local irritation, which causes the removal of “*bad humors*” from “*noble internal organs*” to the place of seton; (2) as a means of preventing “*plague of lungs and spleen*” (as a kind peculiar vaccine in healthy animals), (3) during “*calluses and spongy or fatty growths*” (probably during steatosis) and (4) in animals suffering from long-standing dislocations of joints. Of course, these diseases were not the only ones in which setons were used. Dorohostayski in the book “Hippica…” published in the 17th century, recommended a seton on the neck of horses suffering from deafness [9].

It should be pointed out that setons may be placed on various parts of the animal depending on the animal species and type of disease. In large animals, setons were placed mainly on the neck, back and dewlap (cattle, sheep) or the shoulder, chest, ribs, sacral part of the back, hip, knee and even hoof frog (horses). In dogs, setons were mostly placed on the forehead, temples and neck [12,20,21,23].

Even in the second half of the 19th century setons were very popular. For example, “New surgeon…” (title in Polish: “*Nowy lekarz…*”) written by Jan Mikołaj Rohwles (published in 1872) recommended setons for twenty diseases of horses, cows and dogs, including inflammatory processes in various internal organs (lungs, digestive tract, kidney), neuronal diseases and various infectious diseases [21]. In the second half of the 19th-century veterinarians realized that setons were not effective, but they often recommended this operation, because “*although there is no improvement, setons should be done because they do not require much work or money*” [21].

However, by the turn of the 19th and 20th centuries setons had become obsolete. The book “Knowledge of the treatment of domestic animals” (title in Polish: “*Nauka leczenia zwierząt domowych”)*, published in 1893 recommended setons only in one case, namely, in the treatment of ulcers which could not be cleaned with other methods [15]. In turn, another book, “Popular veterinary for rural householders” (published in the same year) declared that setons were not effective and they were only superfluous cruelty to animals [14]. The same information can be found in books published in the first year of the 20th century [24]. That time marked the end of the use of setons.

### 3.1. “Fonticulus”

“Fonticulus”, also called an aperture, was a type of surgical operation which was similar to the seton. This treatment was used in Polish veterinary medicine (similar to the seton) from the 16th to the end of the 19th century, but it was especially popular in the 18th and the first half of the 19th century [10,12]. “Fonticulus” consisted in an incision in the skin (contrary to setons, where two incisions were made) and the insertion of a leather disk wrapped in a strap under the skin. From time to time, the leather disk was replaced by a rag soaked with turpentine or the root of *Veratrum nigrum* [12]. In earlier centuries (16th and 17th) other things could also be put under the skin. For example, “Hunting with hounds” (1608) recommended the following cure for dogs suffering from paresis: “*cut the skin, separate the skin from muscles with wooden wedges, put lard and pour broken glass in the wound*” [8].

**Figure 5:**
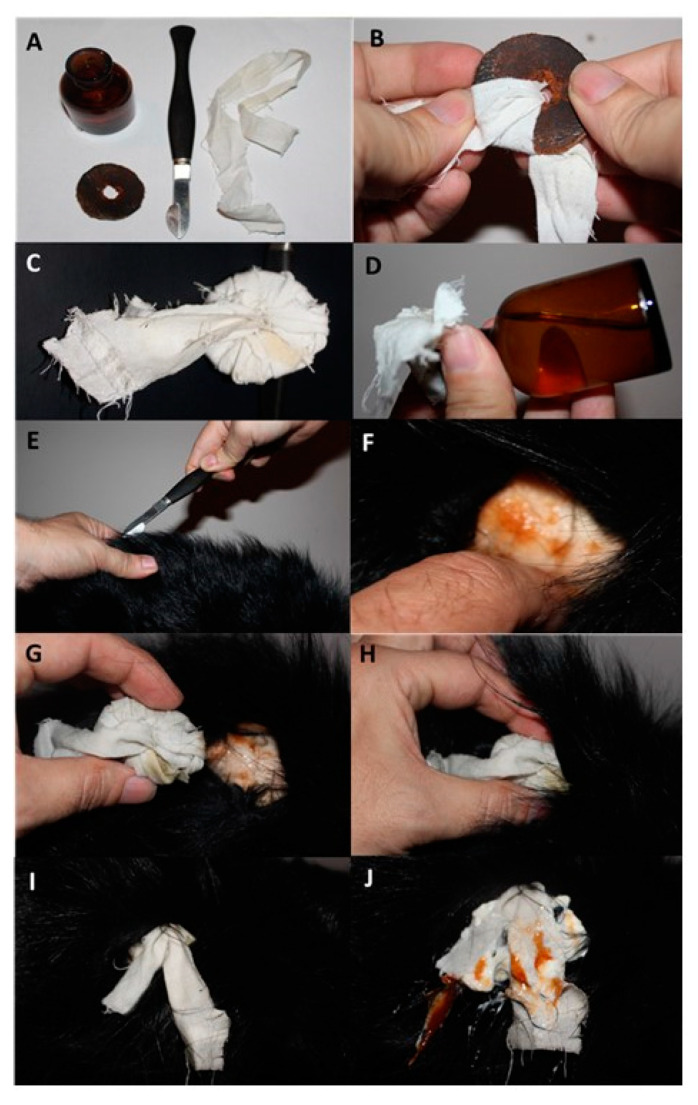
Reconstruction (directed by the authors) of the performance of “fonticulus” according to “Practical veterinary surgery” (1845). (**A**) tools needed to do “fonticulus”: bottle with turpentine, bistoury, leather disk, linen strap; (**B**) wrapping of the leather disk with the strap; (**C**) leather disk ready to use; (**D**) soaking with turpentine; (**E**) incision of the skin; (**F**) separation of the skin from the subcutaneous tissue and muscles; (**G**,**H**) insertion of the disk under the skin; (**I**) “fonticulus” right after the doing; (**J**) “fonticulus” after about 14 days (visible effusion and abscessation of the wound) (the reconstruction was made with food pork meat covered by the lambskin and no animal suffered any damage during reconstruction) (photo. L Rytel).

The exact description and procedure for the “fonticulus” can be found in books published in the 19th century, particularly in “Practical veterinary surgery” (1845) and “Manual of household veterinary medicine” (1858) [12,13]. According to these books, a veterinarian performing “fonticulus” should grab the skin, form a fold and cut the skin with the bistoury. The next stage was the separation of the skin from the muscles around the incision with the finger and the insertion under the skin of a leather disk (three inches in diameter, with a hole in the middle and wrapped in a strap or thread) in such a way that the end of the strap or thread was sticking out of the incision (Figure 5).

The strap or thread was often soaked with irritant substances, including turpentine or Spanish fly (*Lytta vesicatoria*). The leather disk was usually removed from under the skin after 14–24 days. The mechanism of action of “fonticulus” was similar to setons, and both treatments were used in similar diseases. Similar to setons, the use of “fonticulus” in Polish veterinary medicine was discontinued at the end of the 19th century as an ineffective and painful method.

Both setons and “fonticulus” were used in veterinary medicine for ages. As mentioned above, ancient veterinarians believed that these treatment methods were the way to remove “bad humors” from vital organs [12]. Their popularity may indicate that these treatment methods showed some efficacy, but the mechanisms of such efficacy are unknown. Of course, in most cases, setons and “fonticulus” were harmful and painful to patients and even may have contributed to the death of the animal. However, on the other hand, inflammatory and purulent processes accompanying these treatment methods may have contributed to some extent to an increase in immunological system activity. Moreover, the exacerbation of inflammatory processes appearing after treatment with setons or “fonticulus” may have had some positive effects on chronic diseases.

### 3.2. Cauterization

Cauterization in old Polish veterinary medicine was considered one of the most effective methods of treatment. The book “New horse pharmacy” (title in Polish: “*Nowa apteczka końska*”), published for the first time in 1797, states: “*there are no such helpful, brave and universal drugs in horse medicine, as fire*” [25]. Cauterization was considered to be a difficult treatment, because in another place in the book the author wrote: “*A veterinarian who performs cauterization should be skilled and have a light hand*” [25].

For ages, cauterization was recommended during various animal diseases. For example, “The matter of horse medicines…” (1532) for the treatment of strangles in horses prescribed “*burn the skin with a hot iron and then pour over with molten sulfur*” [5]. In turn, Jan Ostroróg in “Hunting with hounds” (1608) recommended “*burn well with a hot iron between the eyes*” in canine rabies and a disease called “*slinogorz*” (probably an inflammatory process within the salivary glands). Moreover, the same book states that cauterization is very helpful for “*bad legs*” (probably movement disorders or lameness) as well as during the treatment of scabs [8]. Cauterization was also very popular in later times. Jakub Haur in “General yeoman economy” (title in Polish: “*Ekonomika ziemiańska generalna*”*)*, a widely read farming handbook, (published for the first time in 1675) described a horse disease called “*tylczak*” (probably a form of glanders) characterized by subcutaneous tumors, in which cauterization is the best treatment [26]. The development of science and modern veterinary in the 19th century did not put a stop to the use of cauterization. Jakub Henryk Lewandowski in the “Manual of household veterinary” (1858) advocated cauterization in the treatment of ulcers, limbs of the horse and warts, as well as during paresis. According to Lewandowski, cauterization should be continued until the skin of the animal turns a dark brown color [13].

**Figure 6:**
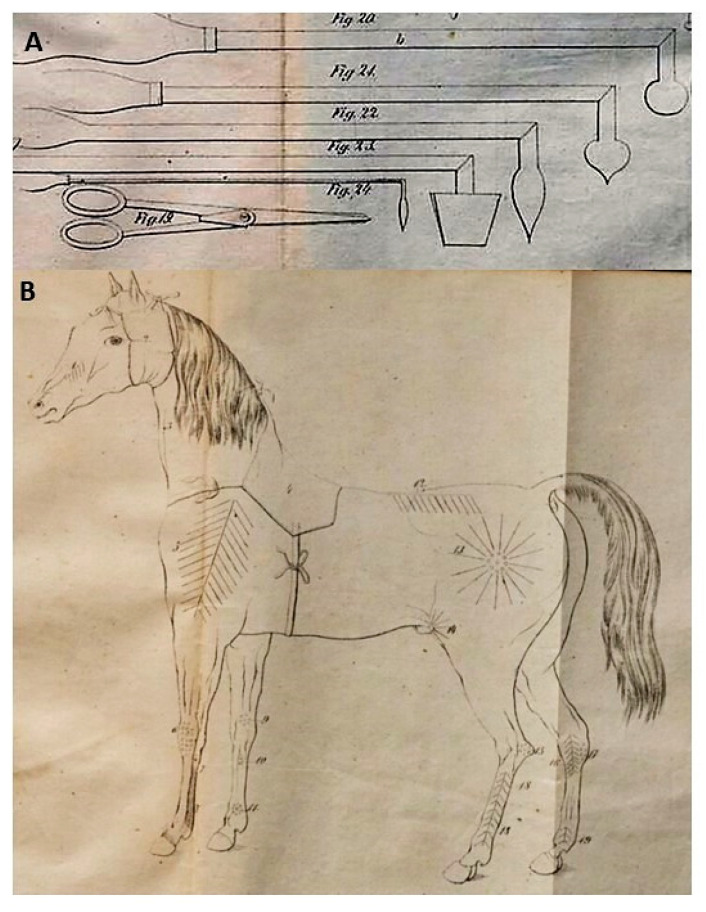
(**A**) Various cautery irons described by Jakub Henryk Lewandowski in “Manual of household veterinary” (1858), (**B**) The illustration from the same book, showing the places and form of cauterization during the treatment in (1) abscesses near teeth, (2) paralysis of the front leg, (3) changes within the knee, (4) induration of tendons, (5,6) “bone circles” (probably any changes on the skin), (7) limbs of the horse on the front leg, (8) paralysis of the spine, (9) paralysis of beck leg, (10) paralysis of the knee, (11) changes on the skin, (12) bone spavin, (13) callus, (14) limbs of the horse on the back leg.

For ages, various equipment was needed for cauterization and various techniques of this treatment were used. Initially (in the 16th and 17th century), simple tools, including hot rods and awls were used [5,9,26]. In books published in the 18th and 19th centuries, specialist tools, known as cautery irons, are described [12,13]. These were the iron rods with a formed metal head with differently shaped and wooden handles (Figure 6A). Different cautery irons were used during different diseases. It should be underlined that cauterization was made on different parts of the animal body, depending on the type of disease [13], as is well illustrated by a figure published in “Manual of household veterinary”, written by Jakub Henryk Lewandowski (1858) (Figure 6B). Sometimes (especially in the 17th and 18th century), a special kind of “internal” cauterization was used. This consisted of the oral administration of irritants. Such curation was recommended by Jan Ostroróg in “Hunting with hounds” (1608) during canine scabs, in which he prescribed sulfur given orally in order to “*burn the decay and bad humidity from the stomach*” [8].

The most comprehensive description of cauterization was shown by Edward Ostrowski in “Practical veterinary surgery” (1845) [12]. Ostrowski described two main types of cauterization. One of them was cauterization with fire, which included: (a) cauterization done with hot cautery irons, (b) treatment with black powder burned on the animal skin (recommended only in field conditions, for example, when dogs became sick during hunting), (c) cauterization with “*moksa*” (the term “*moksa*” meant oakum or linen soaked in flammable liquid, applied to the skin of the animal and burned). The second type of surgery was cauterization with irritants (for example, with acids). Moreover, Ostrowski recommended various types of cauterization during various diseases. In his opinion, even the degree of warming the cautery iron was important. In particular, a red-hot cauterizing iron was used in the treatment of local inflammatory processes, and a white-hot tool was needed for the elimination of “*broken parts of the animal body*” [12].

In the first years of the 20th century, cauterization with fire and burning rods was replaced by the use of irritant substances, such as, for example, Lysol (*Cresolum saponatum*), used in the treatment of ulcers occurring in strangles or “*evil pebble*” (lapis—*Argenti nitras*) used in skin lesion in sheep [16]. The other substances used at that time for such “chemical” cauterization included mustard oil (obtained from White mustard—*Sinapis alba)*, emetic tartar (antimony potassium tartrate), corrosive sublimate (mercuric chloride) and red silver iodide recommended during chronic inflammation and paresis [27].

The popularity of cauterization in ancient veterinary is not clear. Probably this treatment method was considered to be necessary to maintain the balance between internal humors in the living organism. The use of cauterization as a way to clean wounds or remove skin lesions was warranted and achieved good results. However, in many cases (for example, in the treatment of infectious diseases), cauterization only caused unnecessary pain in animals.

### 3.3. Injections

Today it is hard to believe, but in the old veterinary books, intravenous and subcutaneous injections were listed as surgical treatments. Intravenous injections were described as particularly complicated and dangerous operations [12]. Although the first experiments with intravenous administration of various substances including blood, wine, milk and herbal extracts were performed in the 17th century [28], the first descriptions of this treatment in Polish veterinary medicine are from the 19th century. Ostrowski, in “Practical veterinary surgery” (1845), noted that intravenous drug administration may be done only in cases in which oral or anal administration of medicines is not possible (for example, during strong muscle spasms) or in emergencies when drugs must act quickly [12]. According to Ostrowski, to perform intravenous drug administration, the veterinarian had to cut the skin, isolate the vessel, cut its wall and put the horn funnel inside the vein [12]. He should then give the drug by the funnel. Interestingly, for intravenous injections, Ostrowski recommended the same drugs which were administered orally or anally, but in smaller doses. In turn, the agricultural journal “Biblioteka Rolnicza” mentioned that alcohol tinctures of *Aethusa cynapium, Atropa belladonna, Aloe vera, Veratrum album and Strychnos nux-vomica* were most commonly used for intravenous administration [29].

The second type of injections used in old Polish veterinary medicine were subcutaneous injections. They were commonly used in the second half of the 19th century after the invention of the “modern” syringe with a glass barrel and sharp needle by Charles Gabriel Pravaz from 1841–1853 [28]. A wide range of substances were given subcutaneously. The book “Farm veterinary, which is a compendium of knowledge about the treatment of domestic animals” (title in Polish: “*Weterynaria gospodarska czyli nauka leczenia zwierząt domowych*”—printed in 1892) recommended the following drugs for subcutaneous administration [27]:

- Carbonic acid with water and spirit used during bovine miscarriage epidemics;
- Veratrine sulfate and spirit recommended for paresis;
- Pilocarpine chlorate used for indigestion;
- Physostigmine sulfate or apomorphine chlorate used for diseases with cough and tetanus.

## 4. Other Surgical Operations Used in Old Polish Veterinary Medicine

Apart from the surgical treatments mentioned above, a wide range of other operations were used in the history of Polish veterinary medicine. The most interesting and commonly used of them are presented below:

**“*Stinging of mice*”**—the puncture of the parotic glands using an awl during colic in horses [9,30]. It should be pointed out that by the second half of the 18th century “*stinging of mice*” was regarded as an outdated method of treatment. Books published at that period recommended “*breaking up of mice*” instead of stinging [10].

**“*Biting of mice*”**—a variant of “*stinging of mice*”. This method was widely used in the 18th century by peasants and involved the biting of horse parotic glands using their own teeth. The book, “New horse pharmacy…” (title in Polish: “*Nowa apteczka końska*”), published in 1797 states: “*In our country, for the “breaking of mice” the peasants bit them through a handkerchief using their own teeth*” [25].

**The piercing of mares**—this method was used to prevent unwanted mating, in which the labia of the mare was perforated and fastened with special earrings. Veterinary books from the 18th century, state: “*To prevent the pollution of the mare… in inn stables filled with horses… a surgery called piercing should be done. The treatment consists of the perforation of the labia of a mare with a brass steel wire, which should then be bent like an earring*” [10,25].

**The reopening of the urethra in horses**—used in urinary retention. This method consisted of the putting of various objects into the urethra. Dorohostayski in his book “Hippica”, published in the first years of the 17th century, recommended: “*Take a blade of grass, wrap it in hair from the horsetail and stick it into the horse root*” or “*make a thin candle on a string and put this candle in the horse root*” [9]. In turn, in the book “The matter and horse medicines” from 1532, it is stated that one of the best treatments for urinary retention is a living flea inserted in the urethra [5]. Some methods for the treatment of urinary retention were very unusual. For example, the above-mentioned Dorohostayski wrote that a dried bat in a bag suspended on the horse neck had diuretic properties in horses and other species [9].

**“*The cutting of rabies worm*”**—the resection of the vein under the tongue in dogs to prevent rabies. This method was used for centuries. It was described by Jan Ostroróg in the first years of the 17th century (“*A dog has a vein under the tongue. It is good to cut it. Dogs after this cutting do not easily develop rabies*”) [12] and by Krzysztof Kluk in “Wild and domestic animals description…” (title in Polish: “*Zwierząt domowych i dzikich osobliwie kraiowych opisanie…*” published for the first time in 1779) “… *young dogs should be subject to resection of the white thick vein, which is similar to a round, flat worm*” [31]. This method was considered obsolete by the second half of the 19th century.

## 5. Summary

It should be noted that the list of above-mentioned surgical treatments does not include all types of operations performed on animals in the history of Polish veterinary medicine because the diversity of such treatments was enormous. For example, one book alone—“Practical veterinary surgery” (1845) listed more than 90 different surgeries in horses, cattle, pigs, sheep and dogs [12]. Thus, the presentation of all methods used in animal treatment over the centuries in Poland far exceeds the volume of one journal article. In reviewing the methods used in the history of veterinary, not only a huge diversity of utilized methods can be seen, but also the clear evolution of this branch of science. Initially, methods were rather simple and often brutal and connected with superstition. Over time and with the development of knowledge, these methods evolved to more modern treatments. Great progress in veterinary science was made in the 19th century when the basis of modern veterinary medicine was established, and modern methods of animal treatments were introduced.

## References

[1] Perenc A. (1958). History of Animal Health in Poland [Historia lecznictwa zwierząt w Polsce].

[2] Gonkowski S., Felsmann M., Szarek J., Felsmann M. (2013). Treatments of animals in old Polish veterinary [Zabiegi na zwierzętach w dawnej polskiej weterynarii]. Old Medicine and Veterinary.

[3] Janeczek M., Chrószcz A., Ożóg T., Pospieszny N. (2012). History of Veterinary and Deontology [Historia Weterynarii i Deontologia].

[4] Krzyżewski W. About Instruments Using in the Treatment of Animals: Bloodletting Fleam [O Narzędziach Stosowanych w Lecznictwie Weterynaryjnym. Puszczadło do Krwi]. Proceedings of the Lecture delivered during XII Congress of Polish Society of Veterinary Sciences.

[5] Anon (1532). The Matter and Horse Medicines … [Sprawa a lekarstwa końskie].

[6] de’ Crescenzi P. (1542). Books about Holding… [Księgi o Gospodarstwie].

[7] Siennik M. (1564). An Experienced Medicine… [Lekarstwa doświadczone…].

[8] Ostroróg J. (1859). The Hunting with Hounds [Myślistwo z Ogary].

[9] Dorohostayski K. (1620). Hippica, Books about Horses… [Hippika to Jest o Koniach Księgi].

[10] Pietraszkiewicz A. (1754). Horse pharmacy… [Apteczka końska].

[11] Werner J. (1800). The Work for House-Keepers and Peasants… [Dzieło doręczne dla ekonomów...].

[12] Ostrowski E. (1845). Practical Veterinary Surgery [Chirurgia Weterynaryjna Praktyczna].

[13] Lewandowski J. (1858). Manual of Household Veterinary… [Poradnik Weterynaryi Gospodarczej].

[14] Kubicki J. (1893). Popular Veterinary for Rural Householders [Weterynaria Popularna dla Gospodarzy Wiejskich.].

[15] Rohwles J. (1893). The Knowledge about Treatment of Domestic Animals [Nauka leczenia zwierząt domowych].

[16] Steuert L. (1910). Domestic Animal in the Health and Disease [Zwierzę Domowe w Zdrowiu i Chorobie].

[17] Jastrzębiec W. (1945). Veterinary Guide for Farmers and Breeders [Przewodnik Weterynaryjny dla Gospodarzy.

[18] Bolann B., Distante S., Mørkrid L., Ulvik R. (2015). Bloodletting therapy in hemochromatosis: Does it affect trace element homeostasis?. J. Trace Elem. Med. Biol..

[19] Cook L. (2010). Therapeutic phlebotomy: A review of diagnoses and treatment considerations. J. Infus. Nurs..

[20] Mieczyński A. (1875). Setons [Zawłoki]. Bibl. Rol..

[21] Rohwles J. (1872). New Surgeon… [Nowy Lekarz…].

[22] Skrzypek W. (1994). From magic to rational prevention in animal disease [Od magii i wierzeń w zapobieganiu chorobom zaraźliwym do racjonalnej immunoprofilaktyki zwierzęcej]. Med. Weter..

[23] Gonkowski S. (2004). Setons as an old method of animal therapy [Zawłoki jako dawny sposób leczenia zwierząt]. Med. Weter..

[24] Finkel L., Kopia H. (1905). Encyclopedia. Collection from All Branches of Knowledge [Encyklopedia. Kolekcja ze Wszystkich Gałęzi Wiedzy].

[25] Guernire F. (1797). New Horse Pharmacy… [Nowa Apteczka Końska].

[26] Haur J. (1675). General Yeoman Economy… [Ekonomika Ziemiańska Generalna].

[27] Haubner K. (1892). Farm Veterinary, Which Is the Knowledge about Treatment of Domestic Animals [Weterynaria Gospodarska Czyli Nauka Leczenia Zwierząt Domowych].

[28] Myers K. (2009). A history of injection treatments—I the syringe. Phlebology.

[29] Mieczyński A. (1875). Intravenous administration of animal drugs [Dożylne podawanie leków zwierzęcych]. Bibl. Rol..

[30] Olszański Z. Prevention of Inventory Disease [Zapobieganie Chorobom Zwierząt Domowych].

[31] Kluk K. (1809). Wild and Domestic Animals Description … [Zwierząt Domowych i Dzikich Osobliwie Kraiowych Opisanie…].

